# Prevalence and circulant genotypes of *Chlamydia trachomatis* in university women from cities in the Brazilian Amazon

**DOI:** 10.1371/journal.pone.0287119

**Published:** 2024-01-02

**Authors:** Leonardo Miranda dos Santos, Maria Renata Mendonça dos Santos Vieira, Rodrigo Covre Vieira, Lídia Bolivar da Luz Silva, Geraldo Mariano Moraes de Macêdo, Angélica Espinosa Miranda, Danielle Murici Brasiliense, Ricardo José de Paula Souza e Guimarães, Edivaldo Costa Sousa, Stephen Francis Ferrari, Helder Henrique Costa Pinheiro, Edna Aoba Yassui Ishikawa, Maísa Silva de Sousa

**Affiliations:** 1 Tropical Medicine Center, Federal University of Pará, Belém, Pará, Brazil; 2 Institute of Biological Sciences, Federal University of Pará, Belém, Pará, Brazil; 3 Department of Social Medicine, Health Sciences Center, Federal University of Espirito Santo, Vitória, Espirito Santo, Brazil; 4 Section of Bacteriology and Mycology, Evandro Chagas Institute, Ananindeua, Pará, Brazil; 5 Geoprocessing Laboratory, Instituto Evandro Chagas, Ananindeua, Pará, Brazil; 6 Parasitology Section, Instituto Evandro Chagas, Ananindeua, Pará, Brazil; 7 Department of Ecology, Federal University of Sergipe, São Cristóvão, Brazil; Faculdade Sao Leopoldo Mandic, BRAZIL

## Abstract

**Background:**

Approximately 80% of infected women infected by *Chlamydia trachomatis* are asymptomatic, although this infection can lead to serious complications in the female reproductive tract. Few data on *Chlamydia* infection and genotypes are available in Amazonian communities.

**Objectives:**

To describe the prevalence of and associated factors and to identify the genotypes of sexual *C*. *trachomatis* infection in female university students in different urban centers (capital and interiors) in the Brazilian state of Pará, in the eastern Amazon region.

**Methods:**

A cross-sectional study was performed among young women attending public universities in four different urban centers in the eastern Amazon region. They were invited to participate in the studt and cervical secretions were collected for molecular diagnosis of *C*. *trachomatis*. We utilized amplification of the *ompA* gene by nested PCR. Positive samples were genotyped by nucleotide sequencing. Study participants completed a questionnaire on social, epidemiological, and reproductive health variables. A Qui-square and Binominal regression test were used to evaluate the degree of association of these variables with the infection.

**Results:**

A total of 686 female students was included in the study. The overall prevalence of *C*. *trachomatis* was 11.2% (77/686). The prevalence of this infection was higher in interiors (15.2% vs 9.5%/ *p*: 0.0443). Female university students who do not have a sexual partner (11.8%/*p* <0.008), who do not use a condom in their sexual relations (17.8%/*p* <0.0001) and who reported having suffered a miscarriage (32%/*p* <0.0001) have high chances of acquiring this sexual infection. The *ompA* gene was sequenced in only 33 (42.8%) samples, revealing the genotype J was the most frequent (27.2% [9/33]), followed by genotypes D (24.2% [8/33]), and then genotypes F (18.2% [6/33]), E (15.1% [5/33]) K (6.1% [2/33]), Ia (6.1% [2/33]), and G (3.1% [1/33]).

**Conclusions:**

The high prevalence of sexual infection by *C*. *trachomatis* in the female university students from the interior of the state of Pará, individuals with no fixed sexual partner, those that had had a miscarriage, the students that do not use condoms in their sexual relations. The genotype J of *C*. *trachomatis* genotypes was the most frequent. These data are important to help defining the epidemiological effects of chlamydial infections in Amazonian populations.

## Introduction

The *Chlamydia trachomatis* is the most reported sexually transmitted bacterial infection (STI) in the world, remains annually with high infection rates in the United States [1] Europe [2] Africa, and Latin America, including during the period of social isolation of the disease pandemic of 2019 Coronavirus (COVID-19) [1–5]. The analysis of the genetic variability profile of the four variable domains of the *ompA* gene is standard for classifying the 19 genotypes of *C*. *trachomatis* [6]. Genotypes A, B, Ba, and C are related to trachoma [7–9], while genotypes L1, L2, L2a, and L3 are related to lymphogranuloma venereum [10–12]. Genotypes D, Da, E, F, G, Ga, H, I, Ia, J, and K are frequently associated with non-invasive genital infections. In women, 19.7% of that infected progress to a fibrous infectious-inflammatory syndrome known as Pelvic Inflammatory Disease (PID). PID is responsible for 30% of cases of infertility due to tubal factors and 50% of cases of ectopic pregnancies due to tubal occlusion, in addition to being associated with premature birth and spontaneous abortion [13–21].

In Brazil, the absence of a monitoring program contributes to the unawareness of this silent epidemic and its recurrent damages, however, the annual number of hospitalizations of women with PID is over 45,000, however, other factors may be aggravating this situation in the female population living in geographic regions far from the capital in the Amazon, such as the difficulty in accessing specialized health systems, such as specific tests for Polymerase Chain Reaction (PCR), ELISA, resonances between others, as well as lack easy access to treatment regimens and hospitalizations in PID episodes [22]. The studies have reported prevalence of 4% to 20.5% in both asymptomatic women [23–31] and those seeking gynecological treatment [32–34]. The few available studies about genotyping for Brazil have recorded predominant frequencies of genotypes D, E and F of *C*. *trachomatis*, however, this is insufficient to understand what this infection behaves like in Brazil [28, 33].

The female university students is a young female population of reproductive age that often engages in risky sexual behavior, furthermore, that population group in the Brazilian Amazon face a scenario of social vulnerability, in particular remote communities in the deep interior, as they do not have access to the gold-standard laboratory diagnosis for the screening of this asymptomatic infection and thus, prevention of possible late reproductive sequelae [35–42]. The knowledge of the prevalence and circulating genotypes of *C*. *trachomatis* in Amazonian populations can help to understand the epidemiology of this infection and planning prevention strategies focused on these young women. Thus, here, we describe the prevalence of and the associated factors and to identify *C*. *trachomatis* genotypes in university women living in four large urban centers in the Brazilian state of Pará, in the Brazilian Amazon region.

## Methods

### Data collection

The present study, based on a cross-sectional, analytical approach, lasted from February to December 2019. The study involved female students (n = 686) attending different campus from a public university in the Brazilian state of Pará, including 482 students from the state capital, Belém (the capital of the Brazilian state of Pará has a total area: 1,059.466 km^2^, with a total population of 1,499,641 in habitants and a demographic density of 1,315.26 in hab/km^2^, municipal human development index of 0,746) and 204 from campi in three other urban centers in the interior of the state (Altamira [interior, territorial area of 159,533.306 km^2^, estimated population of 117,320 people and a demographic density of 0.62 in hab/km^2^, municipal human development index of 0,665], Bragança [interior 2, territorial area of 2,124.734 km^2^, estimated population of 130,122 people and a demographic density of 54,13 in hab/km^2^, municipal human development index of 0,600], and Castanhal [interior 3, territorial area of 1,029,300 km^2^, estimated population of 205,667 people and a demographic density of 168.29 in hab/km^2^, municipal human development index of 0,673]). All the individuals were invited to participate in the study. Prior contact with the students was supported by collaborators on each university campus, and local radio stations announced the dates and places of sample collection. The exclusion criteria were pregnancy, menstruation, and either not wishing to participate in the study or not signing the informed consent form. The participants were required to fill in a questionnaire and were informed about the importance of providing reliable answers, in order to minimize possible biases in the study. The specific variables investigated in the present study were: age, conjugal status, household income (in multiples of the Brazilian minimum wage), use of tobacco, consumption of alcoholic beverages, age at fist sexual intercourse, lifetime number of sexual partners, current relationship, use of condoms, history of miscarriage, and gynecological disorders. Cervical secretions were collected during routine pelvic examinations using an endocervical brush, and the samples were stored in cryogenic tubes containing 1 ml Tris-EDTA buffer [10 mM Tris-HCl pH 8.5; 1 mM EDTA] at a temperature of -20°C prior to testing.

### Extraction of the DNA

The DNA was extracted using a pureLink Genomic DNA Purification kit (Invitrogen, Carlsbad, CA, USA) according to the manufacturer’s instructions and stored at -20°C until analysis. A Polymerase Chain Reaction (PCR) of the human *β-globin* gene was conducted prior to the detection of *C*. *trachomatis*, in order to confirm the suitability of the samples.

### Detection of the *ompA* gene of *C*. *trachomatis*

The *C*. *trachomatis* DNA sequence was amplified using a nested PCR protocol modified by Jalal et al. (2007) [43], which produced a sequence of 394 base pairs (bps) of the *ompA* gene of *C*. *trachomatis*. The first reaction used 6.0 μL of GoTaq Green Master mix (Promega, Madison, WI, USA), 0.5 μL (20 pmol/μL of each primer) of the primers P1 (A) (5'GACTTTGTTTTCGACCGTGTT-3 ') and P2 (5'AGCRTATTGGAAAGAAGCBCCTAA-3 '), 2 μL of the genomic DNA, and 3 μL of sterile water for a final volume of 12 μL. The second reaction used 0.5 μL of the solution of the first reaction, 6.0 μL of *Go* Taq Green Master mix (Promega, Madison, WI, USA), 4.5 μL of sterile water, and 0.5 μL (20 pmol/μL) of the primers P3 (5'-AAACWGATGTGAATAAAGARTT-3′) and P4 (5'-TCCCASARAGCTGCDCGAGC-3′). In both steps of the nested PCR, negative and positive controls were used to optimize the results. Initial activation was conducted at 95°C in both stages of the PCR, but whereas this temperature was maintained for 5 minutes in the first stage, it was maintained for only 1 minute in the second stage. This activation was followed by 35 cycles of denaturation at 94°C for 40 s, annealing at 54°C for 30s, and extension at 72°C for 90 s, with a final extension step at 72°C for 7 min. The amplified products were visualized by electrophoresis in 1% agarose gel with 0.5 mg/mL of ethidium bromide.

### DNA sequencing

The Sanger method of nucleotide sequencing was used. An approximately 990bp fragment of the *ompA* gene was amplified by nested PCR using primers P1(B) (5′-ATGAAAAAACTCTTGAAATCGG-3′) and OMP2 (5′-ACTGTAACTGCGTATTTGTCTG-3′), and whenever re-amplification was necessary, the inner primers MOMP87 (5′-TGA ACC AAG CCT TAT GAT CGA CGG A-3′) and RVS1059 (5′-GCA ATA CCG CAA GAT TTT CTA GAT TTC ATC-3′) were used [44]. The first step of the nested PCR was run in a 0.5 μL volume containing 20 pmol/ μL of each primer P1(B) and OMP2 and 5.0 μL of the DNA extracted from the endocervical secretion, 14 μL of sterile water, 1.0 μL of MgCl_2_, 1.0 μL deoxynucleoside triphosphate (10mM), 2.5 μL of 10x buffer, and 0.5 μL of Hotstar Taq DNA Polymerase 1.5U (Qiagen). Amplification was run in a final reaction volume of 25 μl [44]. In the two steps of nested PCR a negative and a positive control was used to optimize the result, but these controls were not used in the sequencing.

In the initial step of the nested PCR, amplification conditions were 95°C for 5 min, followed by 40 cycles of 94°C for 30 s, 55°C for 30 s, and 72°C for 90 s, and a final extension at 72°C for 7 min. In the nested PCR, the MOMP87-RVS 1059 primer pair was used with 1.5 μl of the product of the first stage of the nested PCR, which was added to a final volume of 25 μl. The conditions of the second step of the nested PCR were the same as those described above, except for the annealing temperature which was 60°C, and the addition of 17.5 μl of sterile water [44].

The amplified products were visualized by ethidium bromide (0.5 mg/mL) staining after electrophoresis in 1% agarose gel. The products obtained by the nested PCR were purified using a BigDye Xterminator Purification kit (Applied Biosystems, Foster City, CA, USA) to sequence both strands. A BigDyeTerminator Cycle kit (Foster City, CA, USA) was used for the sequencing reaction, according to the manufacturer’s instructions. The reaction mixtures were sequenced in an ABI 3130 (Applied Biosystems, Foster City, CA, USA).

### Genotyping

The sequence obtained of *ompA* gene from the *C*. *trachomatis* was assembled using the CAP3 software, aligned with MAFFT v.7.221 and edited with the Geneious Bioinformatics suite v.8.1.7 and compared with sequences from other studies and available in the GenBank, from the BLAST program of the NCBI (National Center for Biotechnology Information Database) (https://www.ncbi.nlm.nih.gov/) which was also used to the deposit of the sequences obtained in this study. Phylogenetic analysis was performed using maximum likelihood (ML) analysis. Afterwards the phylogenetic reconstruction, also using the software. FastTree was performed using the 1000-replica non-parametric reliability test using the bootstraps method. Finally, the Evolview web server was used to edit the generated phylogenetic tree.

### Ethics statement

This study is part of the project “Detection and genotyping of *C*. *trachomatis* in university students attended at the cytopathology laboratory / UFPA: cytological and molecular analysis”. All methods were performed in accordance with the relevant guidelines and regulations. Our research was authorized by the Research Ethics Committee of the Center of Tropical Medicine, at the Federal University of Pará (process number: 103,571). Only the Belém campus has cytopathology services within the university and the demand for these services is constant, while the countryside campuses do not have this service and it was necessary to make trips (one trip to each campus). The team for this project contacted the coordinators of the campuses in advance to notify the students about the arrival of the respective cytopathology services. As soon as the team arrived at the countryside campuses, new random invitations were made to students through oral invitation and distribution of informational folders. In all cases, capital and interior, only students who read and signed the TCLE were included in this study. All participants were legally over 18 years of age and informed in writing of their free and informed consent for their participation in the study in writing prior to the collection of samples and epidemiological data. All the data were analyzed with complete anonymity. The participants that tested positive for sexual infection by *C*. *trachomatis* were provided counseling and were referred for medical evaluation.

### Statistical analysis

The data were analyzed using the Statistical Package for Social Sciences (SPSS) version 21.0 (SPSS, Chicago, Illinois). The Odds Ratio was used to verify the difference in the prevalence of this infection between the campi of the state capital and the other three urban centers. We pooled the individuals from these localities for the complementary analyses. The degree of association between the prevalence of sexual infection by *C*. *trachomatis* and the study variables were evaluated using Chi-square and Binominal Logistic regressions. The 95% confidence interval (CI) was calculated for these comparisons, and a *p ≤* 0.05 significance level was considered for all analyses.

## Results

A total of 686 university students accepted to participated in the present study. The median age was 25 years (interquartile range: 21.0–29.0 years; amplitude: 18–67 years). The median age of the individuals that tested positive for infection by *C*. *trachomatis* was 23 years (interquartile range: 21.0–29.0 years; amplitude: 18–51 years).

The overall prevalence of sexual infection caused by *C*. *trachomatis* in the study population was 11.2% (77/686; 95% CI: 8.9–13.6%). In Belém, however, the prevalence was lower than this general mean (9.5%), whereas it reached 15.2% on the three interior campi (Table 1).

**Table 1:** Prevalence of sexual infection by *C*. *trachomatis* among university students in the capital and in the three countrys of the state of Pará, Brazil (n = 686).

Cities	Total n = 686 (%)	CT (+) n = 77 (%)		CT (-) n = 609 (%)	OR	IC (95%)	*p value*
					1.6984	1.0422–2.7677	0.0443*
Interiors (Altamira Bragança Castanhal)	204 (29.7)	31(15.2)	(IC 95%: 10.3%-20.1%) ^†^	173 (84.8)			
Capital (Belém)	482 (70.3)	46 (9.5)	(IC 95%: 6.9%-12.2%) ^†^	436 (90.5)			

CT (+): *C*. *trachomatis* positive. CT (-): *C*. *trachomatis* negative. OR: Odds Ratio. 95% CI: 95% Confidence Interval

*: *p value* statistically significant.

^†^ Estimation of a parameter for the prevalence of sexual infection by *C*. *trachomatis*

The bivariate analyses (Table 2) revealed that most of the variables examined did not play a determining (significant) role in the prevalence of sexual infection by *C*. *trachomatis* in the study population. While age was presumed to be a fundamental factor, for example, a slightly higher prevalence of infection (11.7%) was recorded in the older participants (> 25 years old) in comparison with the younger individuals (prevalence = 10.9%), although this difference was not statistically significant (*X*^2^: *p* < 0.05). The prevalence was also higher (but not significantly so) in married students in comparison with unmarried ones (14.8% *vs*. 10.7%), in students with a low household income (11.4% *vs*. 9.1% in high income households), in smokers (20.0% *vs*. 10.9% in non-smokers), in non-drinkers (12.1% *vs*. 10.7% in consumers of alcoholic beverages), in students with a precocious sex life (14.0% *vs*. 10.5% in individuals that initiated activity after 15 years of age), 12.6% (38/301) of those who reported having a current relationship, and in the students with no gynecological disorder (12.4% *vs*. 10.5% in individuals with a disorder). In two cases, however, a significant difference was found. The students that did not use condoms during sexual relations were very significantly (*p* < 0.0001) more prone to infection (17.8% *vs*. 2.0% in condom users), while those that had suffered a miscarriage were similarly more vulnerable to infection (*p* < 0.0001) than the participants that had never had a miscarriage (32.% *vs*. 8.5%).

**Table 2:** Epidemiological characteristics of university students from four cities in the state of Pará, Amazon, Brazil (n = 686).

Variables		Total n = 686 (%)	CT (+) n = 77 (%)	*p-value* (*X*^2^)	Odds Ratio†	IC (95%) †	*p value* †
Age (years)^a^	≤ 25	412 (60)	45 (10.9)	0.8541	0.7872	0.45462–1.3630	0.393
	>25	274 (40)	32 (11.7)				
Conjugal status^a^	Single	605 (88.2)	65 (10.7)	0.3667	1.4472	0.66234–3.1620	0.354
	Married	81 (11.8)	12 (14.8)				
Household income^a^(number of Brazilian minimum wages)	≤1	620 (90.4)	71 (11.4)	0.7095	0.5759	0.22192–1.4943	0.257
	>1	66 (9.6)	6 (9.1)				
Tobacco	Yes	25 (3.7)	5 (20)	0.2743	1.4163	0.40527–4.9492	0.586
	No	661 (96.3)	72 (10.9)				
Álcool	Yes	431 (62.8)	46 (10.7)	0.6384	0.8121	0.47249–1.3960	0.452
	No	255 (37.2)	31 (12.1)				
Age at fist sexual intercourse (years)^b^	≤15	136 (198)	19 (14)	0.3264	0.8540	0.46617–1.5643	0.609
	>15	550 (80,2)	58 (10.5)				
Sexual partner^a^	No	301 (43.8)	38 (12.6)	0.4263	2.1023	1.21412–3.6401	0.008*
	Yes	385 (56.1)	39 (10.1)				
Condom use^a^ ^b^	No	397 (57.9)	71 (17.8)	<0.0001*	12.1164	5.00761–29.3169	< 0.001*
	Yes	289 (42.1)	6 (2)				
Miscarriage ^b^	Yes	78 (11.4)	25 (32)	<0.0001*	5.7833	3.10330–10.7776	<0.001*
	No	608 (88.6)	52 (8.5)				
Gynecological complaints	Yes	491 (71.6)	52 (10.5)	0.4836	0.7170	0.40346–1.2742	0.257
	No	195 (28.4)	25 (12.8)	0.3264			

Variables adjusted to each other in each group—multiple analysis. 95% CI: 95% Confidence Interval.

*: Statistically significant *p value*.

^a^:Current variables.

^b^:Anamnesis variables.

^†^: Binominal Logistic Regression.

The multivariate analyses (Table 2) indicate that the students that do not currently have a fixed sexual partner are more than twice as likely to acquire the infection (Odds Ratio [OR]: 2.1023; 95% Confidence Interval [CI]: 1.21412–3.6401; *p* = 0.008). Similarly, students that have suffered a miscarriage were almost six times more likely to contract sexual infection by *C*. *trachomatis* (OR: 5.7833; [CI95%]: 3.10330–10.7776; *p* < 0.001). However, not using condoms in sexual relations was associated with a 12-fold greater risk of acquiring the infection (OR: 12.1164; [CI95%]: 5.00761–29.3169; *p* < 0.001).

Only 33 (42.8%) of the DNA samples of the 77 positive *C*. *trachomatis* samples were of adequate quality for nucleotide sequencing. Overall (S1 Fig, Table 2), genotype J was the most frequent (27.2% [9/33]), followed by genotypes D (24.2% [8/33]), and then genotypes F (18.2% [6/33]), E (15.1%[5/33]) K (6.1% [2/33]), Ia (6.1% [2/33]), and G (3.1% [1/33]) (S1 Fig).

Most of the sequences amplified (66.6% [22/33]) were obtained from the samples collected on the Belém campus (S2 Fig, Table 2). The majority of the other sequences were from the Castanhal campus (24.2% [8/33), with only one sequences from Altamira (3%[1/33]) and two from Bragança (6%[2/33]) (S2 Fig, Table 3).

**Table 3:** Distribution of the *C*. *trachomatis* genotypes recorded in the samples obtained from female university students on the four campi in Pará state, Brazil.

Campus	Samples genotyped N (%)	Number (%) of individuals with *C*. *trachomatis* genotype (n = 41):						
		J	D	E	F	Ia	K	G
**Total**	33 (100.0)	9 (27.2)	8 (24.2)	5 (15.1)	6 (18.2)	2 (6.1)	2 (6.1)	1 (3.1)
Belém	22 (66.6)	5 (22.7)	7 (31.8)	3 (13.7)	5 (22.7)	0 (-)	2(9.1)	0 (-)
Altamira	1 (3)	0 (-)	0 (-)	1 (100)	0 (-)	0(-)	0 (-)	0 (-)
Bragança	2 (6)	0 (-)	0 (-)	0 (-)	0 (-)	1 (50)	0 (-)	1 (50)
Castanhal	8 (24.2)	4 (50)	1 (12.5)	1 (12.5)	1 (12.5)	1(12.5)	0 (-)	0 (-)

* Number of samples of adequate quality for the sequencing and genotyping of the 990 bp sequence of the *C*. *trachomatis ompA* gene.

## Discussion

The results of this transversal analysis of sexual infection by *C*. *trachomatis* in female university students from four major cities in the Brazilian state of Pará, in the brazilian Amazonia, indicate a general prevalence of 11.2% that was comparable with that recorded in asymptomatic women [23–31] and women seeking gynecological treatment [32–34] of the other regions of Brazil. The prevalence was high in university students from the three campi in the interior of state of Pará.

We believe that what may have contributed to the higher probability of sexual infection by *C*. *trachomatis* to affect university students from the campuses of the three cities in the interior together (15.2% vs. 9.5% [OR: 1.69; 95% CI: 1.04–2.76, p = 0.0443]) is the greatest demand by these young people, who come from small villages and remote communities deprived of a higher education system [45, 46] and primary reproductive health care services, such as clinics and health units and community outreach services, which are services offered only at universities in the urban centers of this study [35–37].

The multivariate analyses indicate that the students that do not have a fixed partner have double the chance of acquiring a *C*. *trachomatis* infection, while those that do not use condoms have a 12-fold greater chance of infection. We feel certain that the students that participated in the present study have some knowledge on the prevention of STI and the importance of using condoms, which they have been exposed to since elementary school [38, 47, 48]. Several factors may nevertheless contribute to a tendency for risk-taking behavior in these students, including their young age, exposure to novel social environments at university, and their propensity for new experiences, which may make them more likely to neglect the need for basic protective measures to ensure their sexual health [39–42].

This scenario is not restricted to the Amazon region, given that similar sexual behavior has been observed in university students from other regions of Brazil [31, 48] and from other countries, around the world [49–51]. Even so, as high rates of sexual infection by *C*. *trachomatis* are typical of the young populations of remote Amazonian communities [28, 34], it would seem reasonable to assume that the findings of the present study represent only the “tip of the iceberg” of the real epidemiological scenario of the infection in this region. We believe that the local scenario is aggravated by the lack of a systematic monitoring program, which would be necessary to prevent the eventual clinical consequences of this silent epidemic. This assumption is supported by the five times greater chance of infection in women who have suffered a miscarriage, which may be mainly a result of the lack of adequate diagnosis and preventive treatment for pregnant women [19, 21]. As Brazil has an excellent unified and decentralized public health system, we believe that the creation of a national program for the screening of sexual infection by *C*. *trachomatis*, integrated into the National Strategic Plan for STDs and AIDS in Brazil, will have a great positive impact on the public health of Brazilian populations, especially if it is aimed at young adults with sexual experience and who are of reproductive age below 24 years of age, since Brazilian studies on the prevalence of this infection indicate a strong association of *C*. *trachomatis* in young people in this age group-age. This will contribute to the identification of asymptomatic cases, which will lead to the formulation of prevention strategies to control and reduce recurrent reproductive harm, such as PID, spontaneous abortion and permanent infertility [23–34, 52]. Sexual infection by *C*. *trachomatis* is a major concern for public health services worldwide. In European countries, the United States, Australia and Japan, official public screening programs have improved the potential for reliable case quantification and allowed public authorities to identify the epidemiological patterns (symptomatic and asymptomatic) of this infection and guide screening strategies, cost-effective treatment and prevention of recurrent late sequelae.

The hospitalization rates and treatment for tubal pathology of infectious origin caused by *C*. *trachomatis* have decreased. There are few Brazilian studies that identify circulating genotypes of *C*. *trachomatis*, however, we recorded considerable variability in the *C*. *trachomatis* genotypes identified in the present study and, overall, similar results have been obtained in other Brazilian studies [28, 33, 50–55] The D-K genotypes of *C*. *trachomatis* does not appear to have any specific distribution pattern or differences in the pathogenicity of their infections, which may be why the genotype frequencies recorded here were similar to those of other populations, around the world [56–59]. The predominance of the D, E, F and J genotypes in genital *C*. *trachomatis* infections may be related to their adaptive capacity in relation to the immunological system of the infected individual, which may also reflect an increased capacity for transmission [60, 61]. Somebody female Amazonian populations is distributed in remote communities that are poorly or irregularly served by public health programs both from the capital and from the interior have precarious social, economic, epidemiological and of displacement conditions, which contribute to the exposure of the resident female populations to STI, which is reinforced by the generally inadequate public health services, situation of geographic isolation and low medical technology, which contribute to the spread of infections by *C*. *trachomatis* [22, 37]. We faced many challenges during the development of the present study, in particular the logistics of sampling relatively remote localities in the interior of the state, which may have often compromised the quality of the biological material. While this limited our capacity to obtain sequences of the *ompA* gene, the findings were broadly consistent with the existing data.

## Conclusions

The results of the analysis of sexual infection by *C*. *trachomatis* in female university students from the Brazilian state of Pará indicated a higher prevalence of infection in students from the interior of the state, in comparison with the capital, individuals with no fixed sexual partner, those that had had a miscarriage, and, principally, the students that do not use condoms in their sexual relations. The genotype J of the *C*. *trachomatis* was the most frequent. These data are important to help defining the epidemiological effects of chlamydial infections in Amazonian populations.

## Supporting information

S1 Fig Results of the phylogenetic analysis of the ompA gene sequences of C. trachomatis detected in the endocervical samples of women from university women from four cities in the State of Pará, Amazonia, Brazil. The samples analyzed in the present study are shown in red, and all others were obtained from GenBank (https://www.ncbi.nlm.nih.gov/genbank).
(TIF)

S2 Fig Prevalence of C. trachomatis genotypes originating from endocervical samples of university students from the four municipalities of the state of Pará, Amazonia, Brazil. (TIF)

S1 Table Accession number of the nucleotide sequences of the C. trachomatis ompA gene identified in this study. (Available at https://www.ncbi.nlm.nih.gov/genbank/).
(DOCX)
