# Supplementary figures and images for: Prevalence and circulant genotypes of Chlamydia trachomatis in university women from cities in the Brazilian Amazon
Source: PLoS One. 2024 Jan 2;19(1):e0287119. doi: 10.1371/journal.pone.0287119 (PMC10760737; doi:10.1371/journal.pone.0287119)

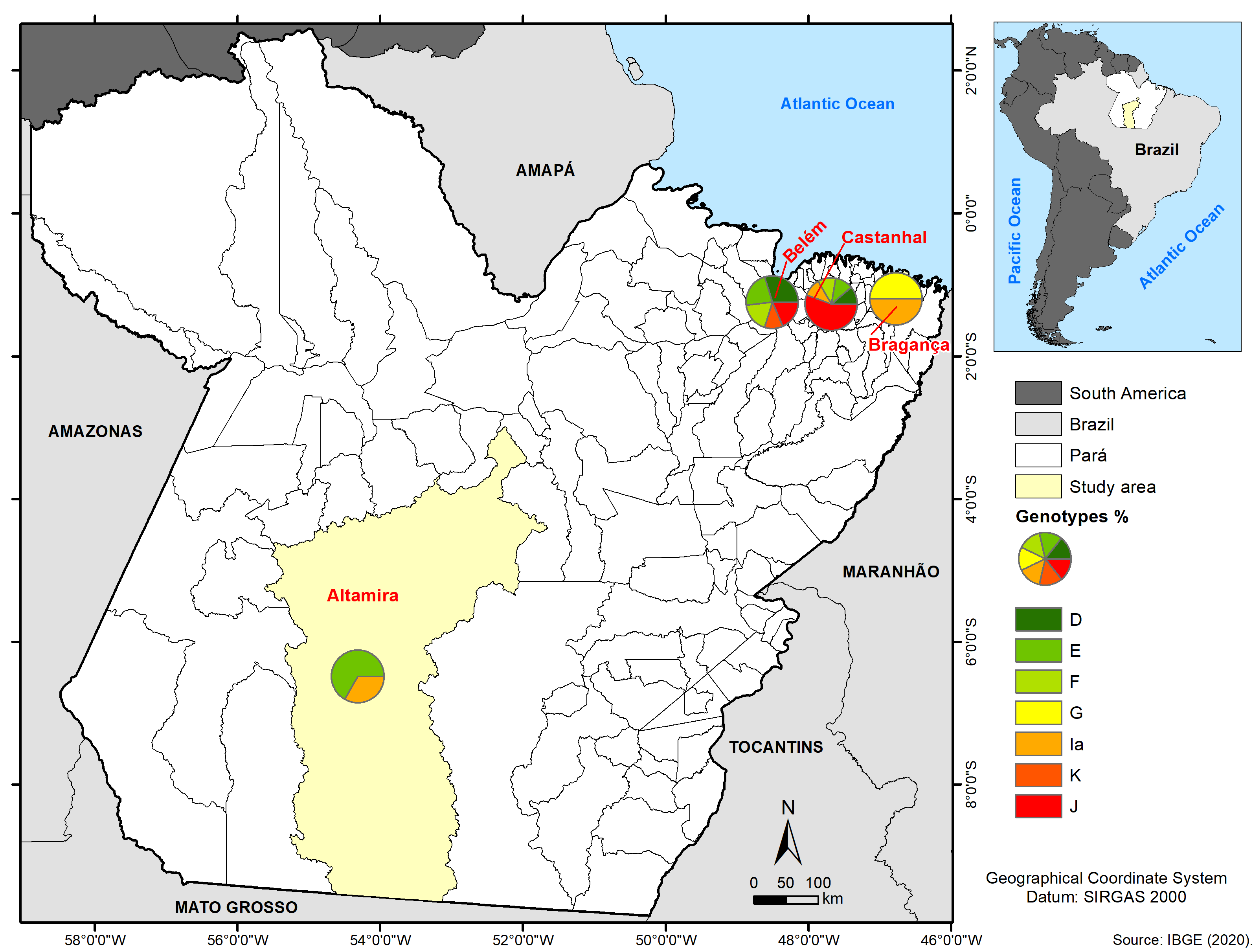

Supplement: S2 Fig — (TIF) [file pone.0287119.s002.tif]
