# Supplementary material for: Prevalence and circulant genotypes of Chlamydia trachomatis in university women from cities in the Brazilian Amazon
Source: PLoS One. 2024 Jan 2;19(1):e0287119. doi: 10.1371/journal.pone.0287119 (PMC10760737; doi:10.1371/journal.pone.0287119)
Supplement: S1 Table — (Available at https://www.ncbi.nlm.nih.gov/genbank/). (DOCX) [file pone.0287119.s003.docx]

| Access Number | Strain | Genotype | County* |
| --- | --- | --- | --- |
| MZ787585 | 249 | J | Belém |
| MK213751 | NMT249 | J | Castanhal |
| MZ787589 | 072 | J | Belém |
| MK213744 | NMT072 | J | Castanhal |
| MZ787586 | 242 | J | Belém |
| MK213750 | NMT242 | J | Belém |
| MZ787587 | 07 | J | Belém |
| MZ787588 | 078 | J | Castanhal |
| MK213745 | NMT078 | J | Castanhal |
| MZ787595 | 015 | F | Belém |
| MZ787594 | 091 | F | Belém |
| MK213746 | NMT091 | F | Belém |
| MK213742 | NMT015 | F | Belém |
| MZ787593 | 10 | F | Belém |
| MZ787592 | 14 | F | Castanhal |
| MZ787598 | 053 | E | Belém |
| MZ787597 | 129 | E | Belém |
| MK213747 | NMT129 | E | Belém |
| MK213743 | NMT053 | E | Castanhal |
| MZ787596 | 37 | E | Altamira |
| MZ787602 | 18 | D | Castanhal |
| MZ787603 | 17 | D | Belém |
| MZ787600 | 48 | D | Belém |
| MZ787604 | 15 | D | Belém |
| MZ787599 | 911 | D | Belém |
| MK213752 | NMT911 | D | Belém |
| MZ787601 | 216 | D | Belém |
| MK213749 | NMT2016 | D | Belém |
| MZ787591 | 04 | G | Bragança |
| MZ787590 | 09 | Ia | Castanhal |
| MK213748 | NMT144 | Ia | Bragança |
| MZ787583 | 46 | K | Belém |
| MZ787584 | 21 | K | Belém |
